# Frequency-Switchable Metamaterial Absorber Injecting Eutectic Gallium-Indium (EGaIn) Liquid Metal Alloy

**DOI:** 10.3390/s151128154

**Published:** 2015-11-06

**Authors:** Kenyu Ling, Hyung Ki Kim, Minyeong Yoo, Sungjoon Lim

**Affiliations:** School of Electrical and Electronics Engineering, Chung-Ang University, Seoul 156-756, Korea; E-Mails: lingkenyunn@hotmail.com (K.L.); muechu@naver.com (H.K.K.); yoomin24@naver.com (M.Y.)

**Keywords:** metamaterial, resonance, microfluidics

## Abstract

In this study, we demonstrated a new class of frequency-switchable metamaterial absorber in the X-band. Eutectic gallium-indium (EGaIn), a liquid metal alloy, was injected in a microfluidic channel engraved on polymethyl methacrylate (PMMA) to achieve frequency switching. Numerical simulation and experimental results are presented for two cases: when the microfluidic channels are empty, and when they are filled with liquid metal. To evaluate the performance of the fabricated absorber prototype, it is tested with a rectangular waveguide. The resonant frequency was successfully switched from 10.96 GHz to 10.61 GHz after injecting liquid metal while maintaining absorptivity higher than 98%.

## 1. Introduction

A metamaterial is an artificial structure composed of periodically arranged metallic patterns and thin wires [1]. Metamaterials can have a broad range of unique behaviors typically not found in nature, such as negative permittivity/permeability and a negative refractive index [2,3]. Because metamaterials have these unusual properties, they have been widely used in microwave and optics applications [4].

Landy *et al*. extended metamaterial applications to a microwave absorber for the first time [1]. Because a metamaterial absorber has the advantages of being very thin and having high absorption, small size, and simple fabrication, many researchers have studied the operation principles of the absorber and improved its performance in microwave, optical, and infrared spectral regions [5,6,7,8]. The metamaterial absorber suffers from a narrow bandwidth because of the characteristics of electric and magnetic resonances. In order to extend the bandwidth, ultra-wideband metamaterial absorbers have been proposed [9,10,11,12]. A frequency-tunable metamaterial absorber can also compensate for bandwidth limitations [13,14]. In addition, the absorber’s frequency tunability can be used for imaging and sensor applications [15,16,17]. Various tuning mechanism, such as optical [18,19], magnetic [20], thermal [21], and mechanical [22] ways, have been applied to frequency-tunable metamaterials. On the other hands, a metamaterial absorber may receive EM waves from arbitrary polarization or incident angles. Therefore, many studies have been carried out for angular- and polarization-insensitive metamaterial absorbers [23,24,25].

Most tunable metamaterial absorbers have been realized by incorporating electronic tuning components. The absorption frequency is continuously controlled by loading varactor diodes between metamaterial unit cells [26]. The relative bandwidth of the metamaterial absorber is extended by 30% because of frequency tunability. Switchable reflectors/absorbers were proposed by connecting PIN diodes to metamaterial unit cells [27,28]. At a specific frequency, the total reflection and total absorption are switched by turning PIN diodes on and off. Microelectromechanical system (MEMS) switches were introduced to realize a switchable metamaterial absorber for the near-infrared spectral region [29]. The absorption ratio is changed by electrostatically actuating metamaterial layers. A frequency-tunable metamaterial absorber was reported by employing graphene wires. The absorber’s frequency tuning range is achieved up to 15% by controlling the Fermi energy of graphene [30]. When an active liquid crystal is incorporated in metamaterial unit cells, the absorption ratio changes by 30%, and the absorption frequency is controlled as well with 4% bandwidth [14]. Recently, microfluidic technology has been applied to a frequency-tunable metamaterial absorber [31]. Its resonant frequency is changed by different dielectric constant of liquids in the channel. Although various tuning techniques have been applied to tunable metamaterial absorbers, the use of liquid metal has not been reported yet. Liquid metal can change not only capacitive components but also inductive components of the unit cell.

In this paper, we propose a novel frequency-switchable metamaterial absorber using liquid metal for the first time. We also demonstrate that liquid metal can be applied to electromagnetic wave applications to achieve frequency switching. The metamaterial unit cell is designed based on the interaction between a metasurface array of highly resonant structures and liquid metal. By injecting liquid metal into microfluidic channels, the absorption frequency can be controlled without applying bias voltages.

In this study, eutectic gallium-indium (EGaIn: 75% Ga, 25% In, by weight) is used as liquid metal and provides advantages over other liquid metals such as mercury [32]. EGaIn has a low viscosity (approximately twice the viscosity of water); therefore, it can be injected into microfluidic channels rapidly at room temperature when pressure is applied to the inlet hole. In addition, EGaIn has a low level of toxicity and has a thin, solid-like oxide skin on its surface to improve mechanical stability (*i.e.*, it is non-volatile). However, mercury has high surface energy, so it is difficult to keep mechanical stability. Therefore, EGaIn is superior to mercury, which is toxic and forms unstable structures. The oxide layer does not grow thicker with time, and therefore, it can maintain its performance in microfluidic channels. In microfluidic applications, EGaIn may have an advantage over molten solders, which require heating and cooling steps that increase the time needed for the fabrication process. Moreover, the temperatures required to melt solder are too high to be compatible with many organic materials. Because of these merits, EGaIn is used as liquid metal for the frequency-tunable metamaterial absorber application.

## 2. Design and Structure

**Figure 1:**
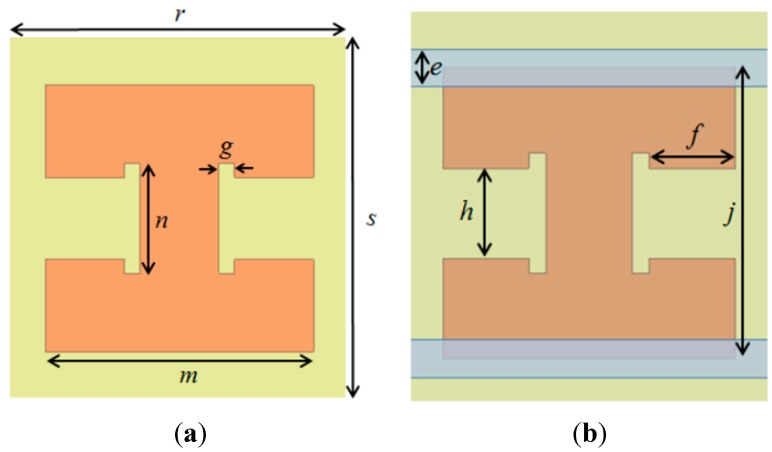
Layout of two metamaterial unit cells: *e* = 0.5 mm, *f* = 1.15 mm, *g* = 0.23 mm, *h* = 1.2 mm, *j* = 3.9 mm, *m* = 3.9 mm, *n* = 1.6 mm, *r* = 4.75 mm, *s* = 5 mm. (**a**) Primitive unit cell; (**b**) Proposed microfluidic unit cell.

Figure 1a shows the top layer of the primitive unit cell. The bottom ground layer is completely covered by copper. Figure 1b shows the top layer of the proposed microfluidic unit cell. In order to generate electric coupling with liquid metal, two microfluidic channels are loaded where the electric field is strongly coupled, as illustrated in Figure 1b.

The resonant frequency of the metamaterial is dependent on both the geometrical dimensions of the conductive pattern and the dielectric constant of substrate. Compared to the air, EGaIn has a higher dielectric constant. When a liquid metal is loaded on the surface of the unit cell, the dielectric constant changes significantly. As a result, the resonant frequency varies depending on the properties of the fluid on the surface. The proposed tuning mechanism is material dependent, while the operation of PIN diodes or MEMS switches are voltage dependent which needs continuous power supply. Although external energy consumption is required to inject liquids into the channels, the microfluidic technology does not require a complex bias network design.

**Figure 2:**
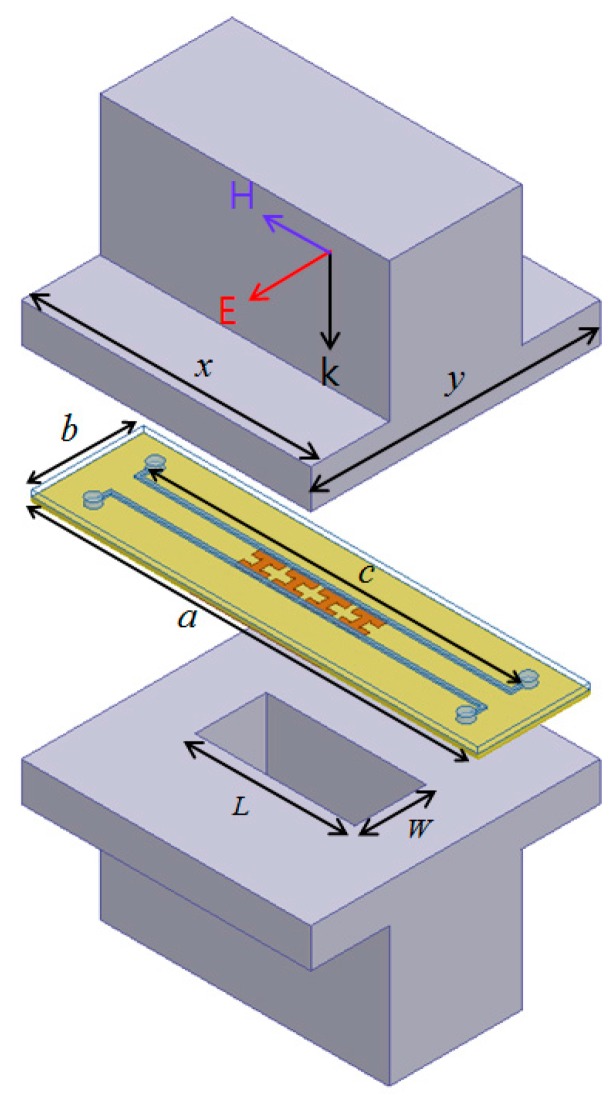
Illustration of the proposed microfluidic metamaterial absorber with the rectangular waveguides: *a* = 64 mm, *b* = 16 mm, *c* = 53 mm, *x* = 41.5 mm, *y* = 41.5 mm, *L* = 22.92 mm, *w* = 10.22 mm.

The absorbing performance of the proposed absorber is tested in the waveguide as illustrated in Figure 2. The proposed absorber is inserted into two open-ended rectangular waveguides in full-wave simulation setup. The incident electric and magnetic fields are indicated in Figure 2. The 1 × 4 unit cell array is designed after considering the size of the waveguide. In order to inject liquid metal through inlets and outlets, we designed the length (a) of the sample to be larger than the length of the waveguide.

**Figure 3:**
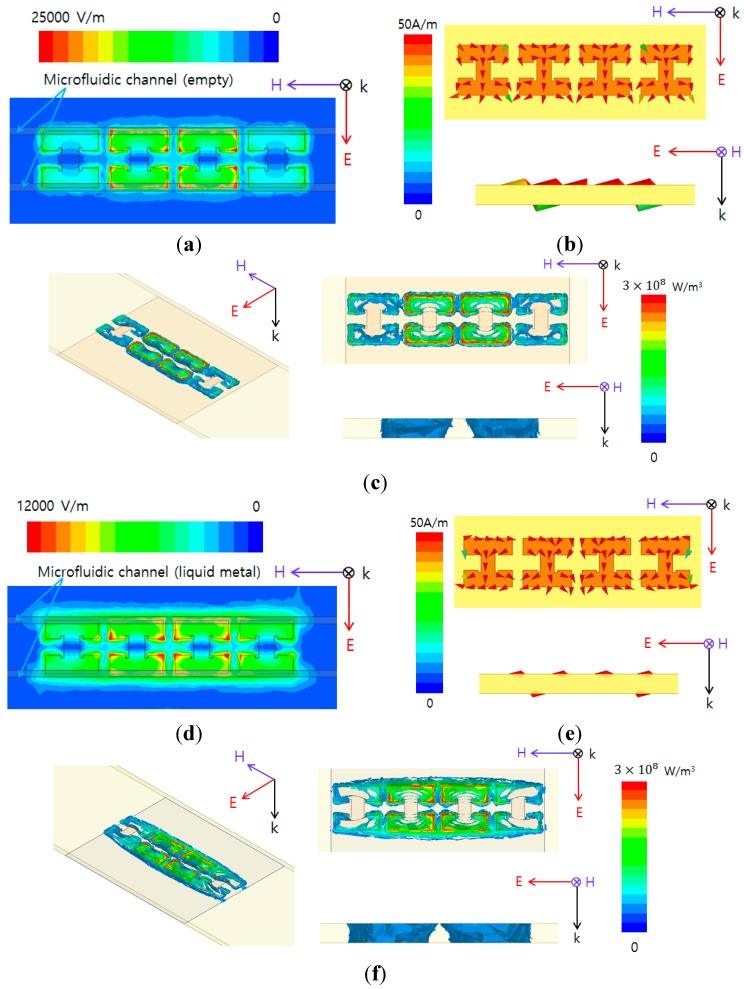
Simulated (**a**) Magnitude of electric field distribution; (**b**) Vector current density; (**c**) Volume loss density at 10.96 GHz when the microfluidic channels are empty Simulated; (**d**) Magnitude of electric field distribution; (**e**) Vector current density; (**f**) Volume loss density at 10.61 GHz when the microfluidic channels are filled with liquid metal.

The absorption phenomenon of the metamaterial absorber can be understood by simultaneous electric and magnetic resonances [33,34]. Figure 3a,d shows the simulated magnitudes of electric fields of the designed microfluidic metamaterial absorber at 10.96 GHz, and 10.61 GHz, respectively. The electric resonance of the proposed metamaterial is generated from the top and bottom layers of a metal structure. In order to control electric resonance, the microfluidic channel is placed on the edges where electric fields are strongly concentrated, as shown in Figure 3a,d. Therefore, strong electric coupling is observed between the conductive pattern and microfluidic channels. This enables the proposed unit cell to be highly sensitive to liquid metal. In order to expound the physical phenomenon, we simulated the surface current distributions of this design. Figure 3b,e shows the simulated vector current densities of the designed microfluidic metamaterial absorber with empty and liquid-metal-filled channels, respectively. Magnetic resonance is observed from the vector current densities of the top and bottom layers that are anti-parallel to each other, as shown in Figure 3b,e. The anti-parallel currents form a magnetic dipole that functions as a current ring. The magnetic dipole direction is along the incident magnetic field polarization. Therefore, it strongly traps the incident magnetic energy, thus resulting in strong absorption. Figure 3c,f shows the simulated volume-loss densities of the designed microfluidic metamaterial absorber with empty and liquid metal-filled channels, respectively. Transmission is minimized by the high loss in the metamaterial, as shown in Figure 3c,f. The total loss can be increased by increasing the substrate thickness. In addition, all the simulated results are plotted by setting the E-field, vector current and volume loss density at peak absorption frequency in full-wave simulation.

Perfect absorption is achieved by zero reflection and transmission coefficients. The reflection coefficient is given by

(1) $$\Gamma \left(\omega \right)=\frac{Z_{0}-Z_{M}\left(\omega \right)}{Z_{0}+Z_{M}\left(\omega \right)}=\frac{\frac{\mu_{0}}{\varepsilon_{0}}-\frac{\mu_{M}\left(\omega \right)}{\varepsilon_{M}\left(\omega \right)}\frac{\mu_{0}}{\varepsilon_{0}}}{\frac{\mu_{0}}{\varepsilon_{0}}+\frac{\mu_{M}\left(\omega \right)}{\varepsilon_{M}\left(\omega \right)}\frac{\mu_{0}}{\varepsilon_{0}}}$$

where *ε*_0_ and *μ*_0_ are the permittivity and permeability of free space, respectively. Therefore, zero reflection is achieved by way of impedance matching between the impedances of metamaterial (*Z*_M_) and free space (*Z*_0_ = 377 Ω). *Z*_M_ can be matched to *Z*_0_ by manipulating effective permittivity (*ε*_M_) and effective permeability (*μ*_M_) of metamaterial. In addition, in order to achieve high absorptivity, a transmitted electromagnetic wave must be lost by dielectric losses, which can be controlled by substrate thickness.

**Figure 4:**
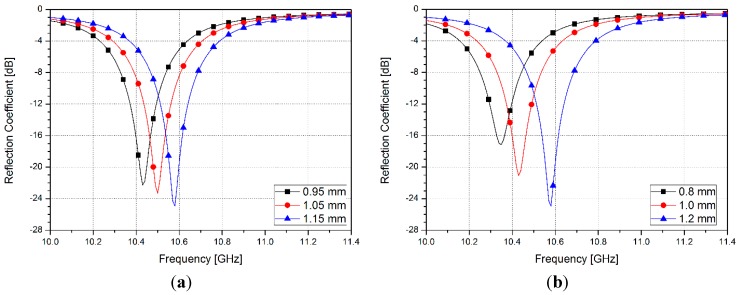
Simulated reflection coefficients of different length of (**a**) *f* and (**b**) *h*.

In order to see the effects of unit cell dimensions, a parametric study has been performed, as shown in Figure 4. Figure 4a shows the simulated reflection coefficients when *f* is 0.95, 1.05, and 1.15 mm. When *f* becomes shorter, the resonance frequency is decreased. In addition, Figure 4b shows the simulated reflection coefficients when *h* is 0.8, 1.0, and 1.2 mm. When the *h* is decreased, the resonant frequency becomes lower.

The complex impedance of the metamaterial absorber is normalized to the impedance of free space. The normalized complex impedance (*z*) of the metamaterial unit cell can be obtained from the S-parameters [35] and is given by

(2) $$z=\sqrt{\frac{{\left(1+S_{11}\right)}^{2}-S_{21}^{2}}{{\left(1-S_{11}\right)}^{2}-S_{21}^{2}}}$$

**Figure 5:**
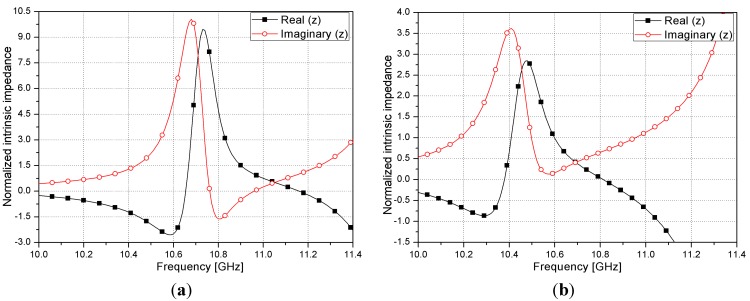
Normalized complex impedance of proposed metamaterial absorber with (**a**) Empty channels; (**b**) Liquid metal-filled channels.

The real and imaginary parts of the normalized impedance are plotted in Figure 5. Figure 5a,b shows graphs with an empty channel and a liquid-metal-filled channel, respectively. It is observed from Figure 5a that the real part is close to one and the imaginary part is approximately zero at 10.96 GHz. Therefore, the absorption frequency at the empty channel state is expected to be 10.96 GHz. Figure 5b shows that the real and imaginary parts are close to one and zero at 10.61 GHz, respectively. Therefore, the absorption frequency at the liquid-metal-filled state is expected to be 10.61 GHz. Consequently, there is no reflection from the boundary between the proposed absorber and air. Because the bottom layer of the proposed absorber is covered with a conductor, the transmission coefficient is zero. Therefore, the proposed absorber possesses high absorptivity at both empty and liquid-metal-filled states at 10.96 and 10.61 GHz, respectively.

## 3. Fabrication and Measurement

**Figure 6:**
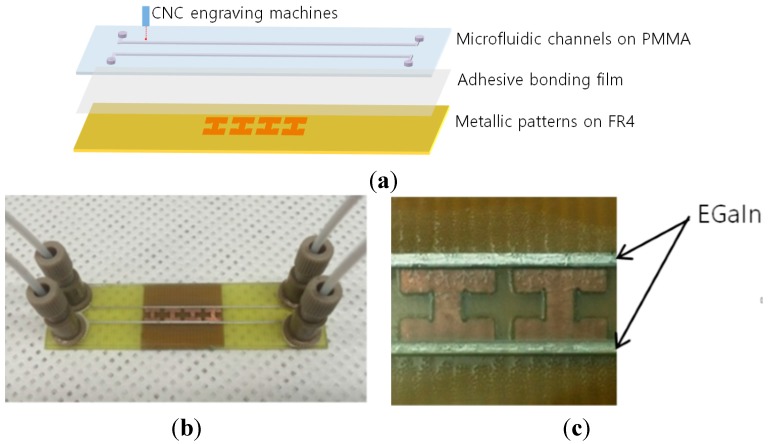
(**a**) Illustration of three layers of proposed microfluidic metamaterial absorber; (**b**) Fabricated absorber sample; (**c**) Zoom-in sample with liquid metal-filled channels.

The proposed metamaterial absorber consists of three layers, as shown in Figure 6a. First, the metallic patterns are fabricated by chemical etching on the FR4 substrate. Next, the microfluidic channels are carved on polymethyl methacrylate (PMMA) using a computer numerical control (CNC) engraving machine, by a process that is simpler and more accurate than laser etching and photolithography. Finally, the FR4 and PMMA substrates are bonded by an adhesive film (ARcare^®^ 92561). The inlet and outlet are realized on PMMA in order to connect tubes. EGaIn is injected in the inlet by a syringe.

In this study, 0.6-mm-thick FR4 substrate is used. Microfluidic channels are engraved on the PMMA substrate. The dielectric constant and loss tangents of FR4 are 4.3 and 0.02, respectively. The dielectric constant and loss tangents of PMMA are 2.55 and 0.002, respectively. Figure 6b shows a picture of the fabricated sample. The zoomed-in images of the empty and liquid-metal-filled microfluidic channels are shown in Figure 6c.

**Figure 7:**
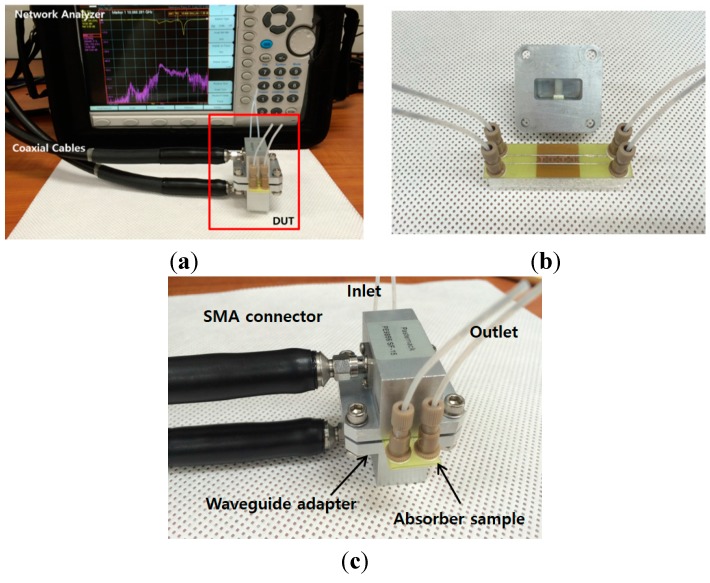
(**a**) Experimental setup to measure S-parameters; (**b**) Open-ended rectangular waveguide; (**c**) The fabricated sample with two SMA-to-waveguide adapters.

To investigate the performance of the proposed metamaterial absorber, the reflection coefficients were measured using a waveguide measurement method. The reflection coefficients can be measured from the S-parameter S_11_. The measurement setup is shown in Figure 7. Absorptivity A(*ω*) can be calculated from reflection coefficients and transmission coefficients. However, the proposed absorber has transmission coefficients of zero because of the back conductive plate [1].

Therefore, the absorptivity is given by

(3) $$A\left(\omega \right)=1-{\left|S_{11}\right|}^{2}-{\left|S_{21}\right|}^{2}=1-{\left|S_{11}\right|}^{2}$$

**Figure 8:**
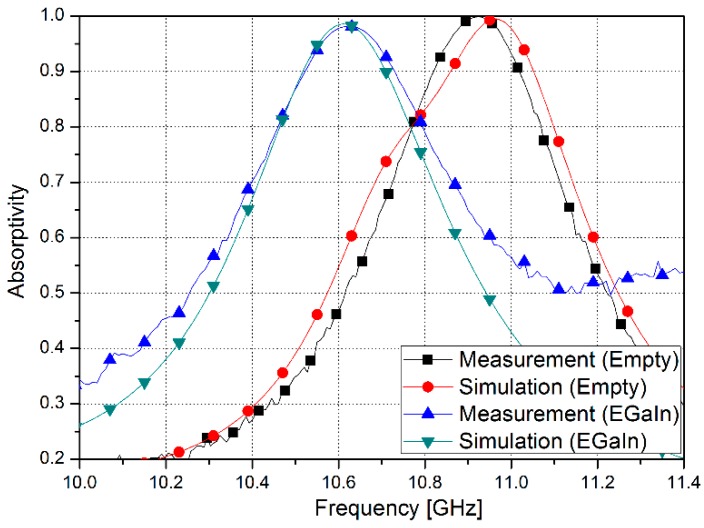
Simulated and measured results for proposed metamaterial absorber sample.

Figure 8 shows the simulated and measured absorptivity of the proposed absorber. Numerical simulation is performed by using the commercial software ANSYS high-frequency structure simulator (HFSS). The reflection and transmission coefficients are simulated using waveguide port excitations of the sample. We set up the boundary condition of the air box as radiation boundaries. The air box size is large enough to ensure the accuracy of simulation.

When the microfluidic channels of the proposed absorber are empty, the metamaterial absorber resonates at 10.96 GHz with 99% absorptivity. When EGaIn is injected into the microfluidic channels of the proposed absorber, the metamaterial absorber resonates at 10.61 GHz with 98% absorptivity. An absorptivity higher than 90% is observed from 10.82 GHz to 11.02 GHz and from 10.52 GHz to 10.73 GHz in the empty and liquid-metal-filled states, respectively. The proposed microfluidic metamaterial absorber successfully achieved both frequency-switching capability and a high absorption ratio.

## 4. Conclusions

In conclusion, we proposed a novel frequency-switchable metamaterial absorber. The frequency-switching capability is achieved by injecting EGaIn into the microfluidic metamaterial unit cells. In order to demonstrate the performance of the metamaterial absorber, a 1 × 4 array sample was fabricated, and full-wave analysis and measurement were performed in a waveguide setup. The resonant frequency was successfully switched from 10.96 GHz to 10.61 GHz after injecting EGaIn. Although the frequency changed, absorptivity was maintained higher than 98% in both the empty and liquid-metal-filled states. Therefore, the proposed metamaterial absorber provided both frequency-switching capability and a high absorption ratio. The proposed metamaterial absorber can be used for large-area sensor applications with micropumps.
